# *KCNQ1* D673N variant causes loss of adrenergic-induced cardiac action potential shortening

**DOI:** 10.1016/j.hrcr.2026.03.020

**Published:** 2026-03-30

**Authors:** Naheed Fatima, Lydia D. Hellwig, Michael G. Klein, Cecelia C. Mangione, Princess Nwachukwu, Joshua Salzer, Clifton L. Dalgard, Joaquin Villar, Mark C. Haigney, Thomas P. Flagg

**Affiliations:** 1 Department of Anatomy, Physiology, and Genetics, Uniformed Services University of the Health Sciences, Bethesda, Maryland; 2 Department of Medicine, Uniformed Services University of the Health Sciences, Bethesda, Maryland; 3 Department of Pediatrics, Uniformed Services University of the Health Sciences, Bethesda, Maryland; 4 The American Genome Center, Uniformed Services University of the Health Sciences, Bethesda, Maryland; 5 Military Cardiovascular Outcomes Research, Uniformed Services University of the Health Sciences, Bethesda, Maryland; 6 Center for Military Precision Health, Uniformed Services University of the Health Sciences, Bethesda, Maryland

**Keywords:** LQTS1, Variant of uncertain significance, Sudden cardiac death, Genetic interpretation, iPSC

**Key Teaching Points**

• Determining pathogenicity of variants of unknown significance in disease-associated genes is an ongoing challenge with implications for the clinical management of patients and their relatives.
• The *KCNQ1* gene variant (c.2017G>A; p. D673N) selectively inhibits adrenergic activation without affecting basal channel functions consistent with a concealed long QT phenotype.
• Functional assessment of *KCNQ1* variants in human-induced pluripotent stem cell–derived cardiomyocytes can provide unique information to aid in variant classification that cannot be obtained using typical recombinant channel systems.

## Introduction

Approximately 350,000 people in the United States experience an out-of-hospital cardiac arrest or sudden death annually, of which approximately 90% are fatal.^1^ Most cardiac arrests occur in an older population with coronary artery disease, but events can also occur in young and otherwise healthy individuals.^2^ In individuals younger than 40 years, the incidence is estimated to range from 4 to 14 per 100,000 person-years worldwide.^3^ In this population, the most common underlying etiologies include arrhythmias and cardiomyopathies, which can be acquired or inherited.^4^ Cardiac evaluation and genetic testing are recommended for young individuals who experience a sudden cardiac arrest.^5^ Although genomic testing capabilities have improved and cost of sequencing has decreased over time, interpretation of genomic information remains challenging.^6^ Functional studies are needed to advance understanding of the molecular mechanisms of disease and help characterize specific variants.^7^

We present findings from an in vitro electrophysiological approach to a rare *KCNQ1* variant of uncertain significance (VUS) identified in a 39-year-old male who survived a sudden cardiac arrest after completion of a half marathon. Loss-of-function mutations in *KCNQ1* are associated with long QT syndrome type 1 (LQTS1) and increased likelihood of developing torsades de pointes ventricular tachycardia.^8^^,^^9^ We show that the variant has no effect on channel function under basal conditions, but markedly inhibits the *β*-adrenergic activation of slow delayed rectifier potassium current (I_Ks_) and consequent shortening of the cardiac action potential (AP) in human-induced pluripotent stem cell (hiPSC)–derived cardiomyocytes (hiPSC-CMs) harboring the mutation. The results suggest that this rare variant could be associated with a concealed LQTS1 phenotype that may have contributed to the sudden cardiac arrest in this patient.

## Methods

### Genomic sequencing

This individual was enrolled in the Genetic Exploration of Military Sudden Cardiac Arrest Study. This study was reviewed and approved by the Uniformed Services University Institutional Review Board (MED-83-8797). The individual was found to be heterozygous for a VUS in *KCNQ1:* c.2017G>A. This variant was also identified and classified as a VUS through clinical genetic testing at GeneDx Laboratory via the combined cardiac sequencing and deletion/duplication panel of 139 genes.

### In vitro functional studies

Plasmids carrying *KCNQ1* (Addgene; #173161) and *KCNE1* (Addgene; #173160) were gifts from Al George.^10^^,^^11^ The D673N missense variant was introduced into the *KCNQ1* complementary DNA using overlap extension polymerase chain reaction. The VUS (*KCNQ1:* c.2017G>A) was introduced in the ND2.0 hiPSC line (a gift from Dr Jizhong Zou, director, National Heart, Lung, and Blood Institute iPSC Core) using CRISPR-Cas technology. To obtain ventricular-type cardiomyocytes from hiPSCs, we used a method adapted from Lin et al.^12^ Electrophysiological function was assessed using whole cell perforated patch clamp experiments in individual cells or in hiPSC-CM monolayers plated on multielectrode arrays (Axion Biosystems) before (control) and 60–90 minutes after drug addition. All experiments were conducted at 35°C–37°C. HMR1556, ML277 (Tocris/Bio-Techne, Minneapolis, MN), and isoproterenol (Sigma-Aldrich) were applied at concentrations indicated. See the Supplemental Material for more details (Supplemental Methods).

### Data and statistical analysis

Data are reported as means ± standard error of the mean or standard deviation. The normality of sample distributions was determined using the Shapiro-Wilk test. Statistical differences between means were determined using a 2-tailed paired or unpaired *t* test for comparisons between groups. *P* < .05 was considered statistically significant.

## Results

### Clinical presentation

**Figure 1:**
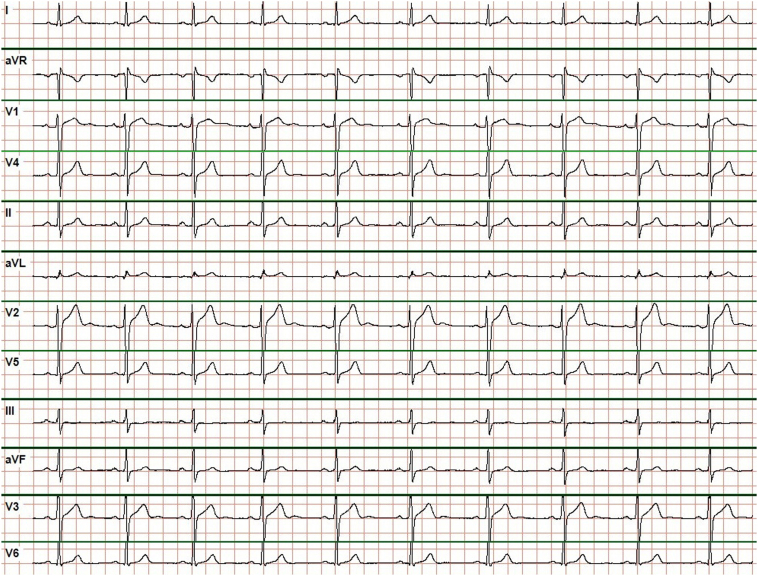
12-lead electrocardiogram after sudden cardiac arrest. The tracing shows a normal sinus rhythm at 60 beats per minute and a corrected QT interval of 400 ms with no evidence of previous myocardial infarction, Wolff-Parkinson-White syndrome, arrhythmogenic right ventricular cardiomyopathy, left ventricular hypertrophy, type 1 Brugada syndrome, or long QT syndrome.

A 39-year-old man experienced a sudden cardiac arrest after finishing a half marathon. The individual reported feeling lightheaded and having tunnel vision before falling and losing consciousness. He denied any chest pain or palpitations associated with the event. The individual received 8 minutes of cardiopulmonary resuscitation and 2 automated external defibrillator shocks. Automated external defibrillator interrogation confirmed ventricular fibrillation. After the event, the patient was admitted for cardiac workup. His echocardiogram showed a normal ejection fraction and mild right atrial dilation. Computed tomography angiogram showed an absence of coronary heart disease and normal origins of the coronary arteries. The 12-lead electrocardiogram was normal with no evidence of left ventricular hypertrophy, Brugada syndrome, or long QT syndrome (LQTS) (Figure 1). An exercise stress test was completed where the patient exercised for 12 minutes and achieved 169 beats/min. The individual had a normal baseline corrected QT (QTc) interval of 430 ms, rare monomorphic PVCs at peak exercise, no ischemic electrocardiographic changes, and a 1-minute recovery QTc interval of 398 ms and a 4-minute recovery QTc interval of 427 ms. An implantable cardioverter-defibrillator was implanted, and the individual was referred for genetic evaluation.

### Clinical genetics interpretation of *KCNQ1* c.2017G>A

Genetic analysis identified a rare *KCNQ1* variant (*KCNQ1*, NM_000218.2: c.2017G>A [p.D673N]) in the individual. KCNQ1 is a major component of the slow delayed rectifier potassium current (I_Ks_) associated with cardiac AP repolarization.^13^^,^^14^ This variant was identified in 10 of 186,262 chromosomes in the general population by the Genome Aggregation Database and has been reported by multiple submitters in ClinVar as a VUS with no conflicts (ClinVar variation ID: 450725). Computational prediction tools suggest that this variant may not affect protein structure and function (rare exome variant ensemble learner score ≤0.5).^15^ There have been no published functional studies of the KCNQ1 D673N variant; however, a patient with this variant was diagnosed as having hypertrophic cardiomyopathy at 15 years of age and died at 23 years of age.^16^

### *KCNQ1* D673N is indistinguishable from wild type under basal conditions

**Figure 2:**
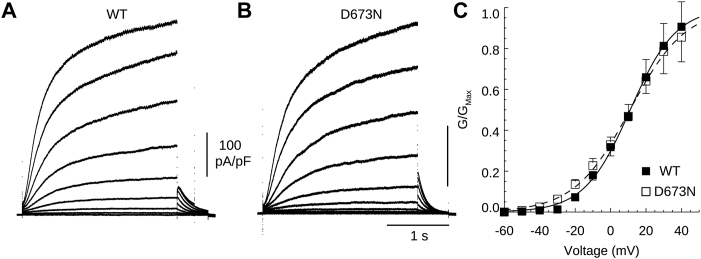
*KCNQ1* D673N substitution does not affect KCNQ1-I_Ks_ current amplitude or voltage sensitivity. Cells were transfected with either WT KCNQ1 (**A**) or D673N KCNQ1 (**B**), plus KCNE1 and GFP, and subjected to a family of increasing depolarizations from the holding potential of −60 mV, followed by a repolarizing step to −50 mV. **C:** The conductance vs voltage relationships for WT (n = 7 cells) and D673N (n = 7) show no significant differences in voltage activation. Points are means ± SEM of the current at the end of the test pulse. The lines are a fit of the data to a Boltzmann function, 1 / (1 + exp[V_1/2_ – V] / k). WT: V_1/2_ = 11.1 ± 3.4 mV, k = 12.9 ± 2.1 mV. D673N: V_1/2_ = 10.9 ± 5.1 mV, k = 15.7 ± 2.7 mV. GFP = green fluorescent protein; I_Ks_ = slow delayed rectifier potassium current; SEM = standard error of the mean; WT = wild type.

To assess the effect of the variant on KCNQ1 channel function, we first engineered a complementary DNA construct harboring the D673N mutation for heterologous expression in HEK293 cells. To recapitulate I_Ks_,^13^^,^^14^ wild-type (WT) or mutant (D673N) channels were expressed with the KCNE1 accessory subunit, and current was assessed in whole cell voltage clamp experiments. There were no differences in peak current, steady-state activation, voltage dependence, or steepness as determined from conductance-voltage relationships (Figure 2). Similarly, no significant differences were observed in cells expressing WT or mutant KCNQ1 without KCNE1 (Supplemental Figure 1). Based on these results, the D673N mutation has no measurable effect on channel assembly, function, or trafficking.

**Figure 3:**
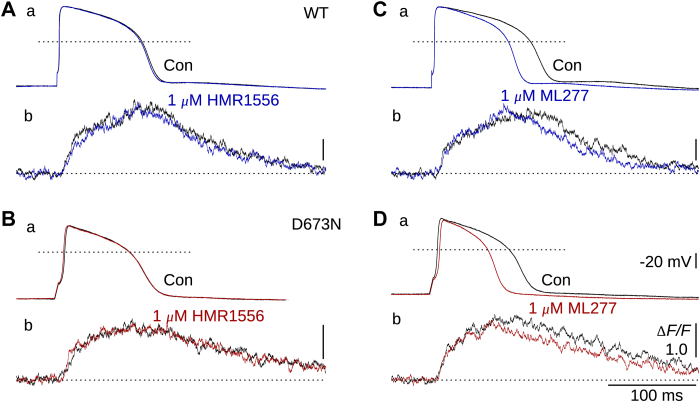
Action potentials (APs) and Ca_T_ are not different in WT and mutant iPSC-derived cardiomyocytes. Shown are representative AP (top) and Ca_T_ (bottom) in (**A**) WT or (**B**) D673N mutant iPSC-derived cardiomyocytes before (Con, *black*) and during exposure to HMR1556 (1 μM). Exposure to ML277 (1 μM) in both WT (**C**) and D673N cells (**D**) exhibited a marked reduction of the AP and Ca_T_ duration. Ca_T_ = calcium transient; iPSC = induced pluripotent stem cell; WT = wild type.

To assess the effect of the variant on cardiac myocyte function, we engineered a human iPSC line harboring the *KCNQ1* c.2017G>A variant and measured cellular APs and myoplasmic calcium transients (Ca_T_) in individually paced hiPSC-CMs. The introduction of the D673N variant did not affect the cellular AP duration (APD) or triangulation (Table 1). A small increase in mean diastolic voltage probably led to a significant increase in amplitude and upstroke velocity. Brief exposure to HMR1556 (1 μM), a specific KCNQ1/I_Ks_ blocker, had no significant effect on either APD or Ca_T_ compared with pre-exposure control in either WT or D673N cells (Figure 3A and 3C). Exposure to the specific KCNQ1/I_Ks_ activator, ML277 (1 μM), resulted in marked shortening of APD at 90% repolarization and concomitant reduction of peak and half-width of the Ca_T_ in both WT and mutant cells (Figure 3B and 3D).

**Table 1:** Baseline action potential characteristics in WT and D673N iPSC-derived cardiomyocytes

Condition	n	APD90 (ms)	APD90/APD30	Diastolic (mV)	Amplitude (mV)	V_max_ (mV/ms)
WT	49	67.8 ± 3.4	1.54 ± 0.02	−46.2 ± 0.9	82.1 ± 1.5	18.6 ± 1.0
D673N	41	63.7 ± 3.9	1.50 ± 0.09	−49.2 ± 1.2	90.6 ± 1.9	25.0 ± 1.7
*P* value		.419	.386	.046	<.001	.001
Effect size (95% CI)∗		0.172 (−0.243 to 0.581)	0.184 (0.231–0.599)	0.427 (0.008–0.847)	0.755 (0.326–1.185)	0.714 (0.286–1.142)

APD_30_ = action potential duration at 30% repolarization; APD_90_ = action potential duration at 90% repolarization; CI = confidence interval; iPSC = induced pluripotent stem cell; V_max_ = peak depolarization rate; WT = wild type.

∗ Effect size calculated as Cohen’s d.

**Figure 4:**
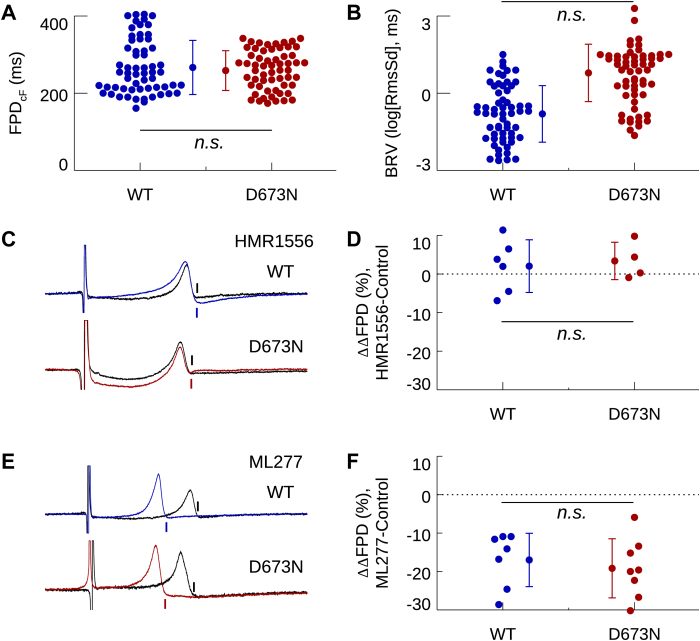
Characterization of iPSC cardiomyocytes in MEA recordings. FPD_cF_ (Fridericia correction) (**A**) and BRV (log[root-mean-square of successive differences]) (**B**) recorded from WT (*blue*) or D673N (*red*) cell monolayers plated in individual wells of 5 plates. **C and E:** Representative field potential recordings from WT or D673N before or during exposure to the I_Ks_ inhibitor, HMR1556 (0.5 μM), or the I_Ks_ inhibitor, ML277 (0.5 μM). **D and F:** Sample distributions of all wells tested expressed as the ΔΔ% difference between exposure and pre-exposure. Mean ± SD of each distribution is indicated. No significant differences between WT and D673N lines were observed. BRV = beat rate variability; FPD = field potential duration; I_Ks_ = slow delayed rectifier potassium current; iPSC = induced pluripotent stem cell; MEA = microelectrode array; n.s. = not significant; SD = standard deviation; WT = wild type.

We also used microelectrode arrays to measure field potentials generated in WT and mutant iPSC-CM monolayers (Figure 4). Neither the field potential duration (FPD) (Fridericia correction) nor beat rate variability was significantly different between the WT and D673N cells. Consistent with the results in individual cardiomyocytes, exposure to HMR1556 (0.5 μM) had no significant effect on FPD, whereas treatment with ML277 (0.5 μM) significantly shortened the FPD in both WT (−16.9% ± 2.8%; *P* < .001) and D673N (−19.1% ± 2.4%; *P* < .001). These results indicate that I_Ks_ is expressed in but contributes little to shaping the AP under basal conditions. Activation of I_Ks_ with ML277 shortens the APD and FPD as expected, but there are no differences between WT and D673N cells.

### *KCNQ1* D673N variant inhibits adrenergic-dependent AP shortening

**Figure 5:**
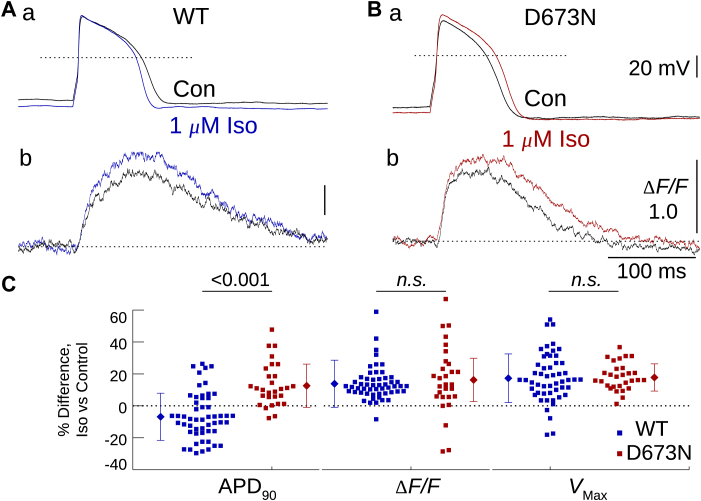
Iso-induced APD shortening is absent in cells harboring the KCNQ1 D673 variant. **A and B:** Representative action potential (top) and Ca_T_ (bottom) recordings before (*black*) and during exposure to Iso (1 μM) in WT (*blue*) and D673N mutant (*red*) iPSC-derived cardiomyocytes. **C:** Summary data from experiments in panels A and B. Iso induced a significant shortening of the APD_90_ in WT but a marked prolongation of the APD_90_ in mutant cells. This is not likely caused by a general loss of Iso sensitivity given that the Ca_T_ amplitude (ΔF/F) and V_max_ were similarly increased in both, consistent with Iso-dependent increase in I_Ca,L_. APD = action potential duration; APD_90_ = action potential duration at 90% repolarization; Ca_T_ = calcium transient; Con = control; iPSC = induced pluripotent stem cell; Iso = isoproterenol; I_Ca,L_ = L-type calcium current; V_max_ = peak depolarization rate; WT = wild type.

Physiological activation of the sympathetic nervous system increases both I_Ks_ and L-type calcium current (I_Ca,L_) in cardiomyocytes with the net outcome of shortening the cardiac AP and potentiating the Ca release from the sarcoplasmic reticulum. Stimulation of the cells with a *β*-adrenergic receptor agonist unmasked a marked difference between the WT and mutant cells (Figure 5). Exposure to isoproterenol (1 μM) caused a significant shortening of the APD at 90% repolarization in WT cells (−6.9% ± 2.0%; n = 53; *P* = .001), consistent with activation of I_Ks_. Isoproterenol also caused an increase in the Ca_T_ amplitude (13.8% ± 1.5%; n = 53; *P* = .001) as expected with activation of I_Ca,L_. In iPSC-CMs, I_Ca,L_ also contributes significantly to the upstroke of the AP owing to the partially depolarized diastolic potential and consequent suppression of the sodium current. Thus, potentiation of I_Ca,L_ by isoproterenol is reflected in the increase of peak depolarization rate (17.3% ± 2.1%; n = 53; *P* = .001). In cardiomyocytes expressing the KCNQ1 D673N mutant, isoproterenol caused a similar increase in both Ca_T_ amplitude (16.2% ± 3.9%; n = 53; *P* < .001) and peak depolarization rate (17.8% ± 1.6%; *P* < .001), demonstrating that *β*-adrenergic receptor engagement activated I_Ca,L_ in the mutant cells. In contrast, isoproterenol exposure caused a marked prolongation of the AP in D673N mutant cells (12.6% ± 2.5%; *P* < .001), suggesting that *β*-adrenergic receptor stimulation failed to activate I_Ks_ containing the KCNQ1 D673N mutant.

**Figure 6:**
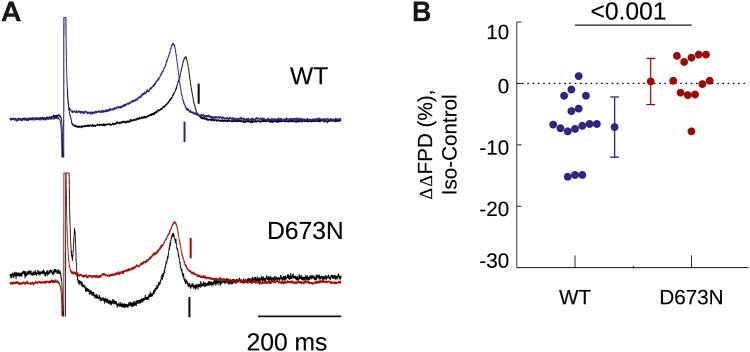
Isoproterenol-induced FPD shortening is abrogated by the KCNQ1 D673 variant. **A:** Representative field potential recordings in WT and D673N iPSC-derived cardiomyocyte monolayers before (*black*) and during (*blue*, *red*) exposure to isoproterenol. **B:** Summary data from experiments in panel A. FPD = field potential duration; iPSC = induced pluripotent stem cell; WT = wild type.

Field potential measurements in cardiomyocytes revealed a similar phenotype (Figure 6). Activation of *β*-adrenergic receptor-dependent I_Ks_ with isoproterenol (0.1 μM) induced a marked shortening of the FPD in WT cells (−7.1% ± 1.2%; *P* < .001). In contrast, the FPD was unchanged in the presence of isoproterenol in hiPSC-CM monolayers harboring the D673N mutant (0.3% ± 1.1%; *P* = not significant). Taken together, these results indicate that the KCNQ1 D673N variant selectively abrogates adrenergic-dependent activation of I_Ks_ and the normal shortening of the AP associated with adrenergic stimulation without effect on the increase in I_Ca,L_ and Ca release from the sarcoplasmic reticulum.

## Discussion

Sudden cardiac arrest is a rare occurrence in young (<40-year-old) individuals and is often associated with inherited cardiomyopathy, including LQTS.^4^ Clinical assessment of the patient revealed no functional or structural cardiac deficits and no evidence of a prolonged QTc interval, but genetic analysis revealed a rare VUS in the *KCNQ1* gene. Advances in genomic sequencing technologies have markedly improved the capability to identify genetic variants, and more than 2000 *KCNQ1* variants have been reported in ClinVar.^17^ Importantly, however, the ability to clinically interpret identified genetic variants continues to be a challenge.^18^, ^19^, ^20^ Identification of pathogenic variants in genes associated with LQTS can help with risk stratification and clinical management of patients and their relatives.

The American College of Medical Genetics and Genomics standards and guidelines for interpretation of sequence variants include consideration of functional evidence.^21^ Variant frequency, structural predictions, computational approaches, and functional assessment have all been used to aid in determining variant pathogenicity.^22^, ^23^, ^24^, ^25^, ^26^, ^27^ The functional consequence of a specific variant can be hard to determine. In the case of the variant described here, the location of the missense mutation is located in a region of the protein that is not included in structural models and has not previously been associated with any critical channel functions, and we found that the mutant channel is indistinguishable from the WT under basal conditions. This does not rule out a pathogenic role of the mutation, but suggests that the phenotype may be concealed as a result of variable penetrance or expressivity.^28^

Arrhythmic events in patients with LQTS1 are often precipitated by stress or physical activity, suggesting that the electrical sequelae of sympathetic stimulation may unmask an LQTS phenotype.^9^^,^^29^ Indeed, the patient described in the present study suffered cardiac arrest after completing a half marathon. Adrenergic activation of I_Ks_ requires the interaction of a macromolecular signaling complex with the COOH-terminus of the KCNQ1 channel.^30^^–^^32^ Our results show that isoproterenol-induced AP and field potential shortening is abrogated when KCNQ1 D673N is expressed in iPSC-CMs, consistent with the conclusion that the D673N variant interferes with the assembly of this macromolecular complex thereby inhibiting I_Ks_ activation by adrenergic stimulation similar to what has been shown for the KCNQ1 G589 variant.^31^^,^^32^

The loss of adrenergic-induced AP shortening would not have been observed if we had only focused on studying recombinant channels in HEK293 cells. iPSC-CMs are increasingly recognized as important tools for understanding patient-specific phenotypes and assessing channel functionality impacts of specific genetic variants.^33^, ^34^, ^35^ In the clinical care setting, a comprehensive multidisciplinary approach is recommended for the evaluation and management of individuals and families with cardiogenetic conditions.^36^^,^^37^ Findings from this study may support expansion of this multidisciplinary approach to include experts in electrophysiological functional studies. Furthermore, the integration of an electrophysiological approach using iPSC technology in clinical cardiogenetics may offer additional opportunities to better tailor management and therapy choices.^38^

## Disclosures

The authors have no conflicts of interest to disclose. The opinions and assertions expressed herein are those of the authors and do not reflect the official policy or position of the Uniformed Services University of the Health Sciences or the Department of Defense and the Henry M. Jackson Foundation for the Advancement of Military Medicine, Inc.
