# Supplementary material for: KCNQ1 D673N variant causes loss of adrenergic-induced cardiac action potential shortening
Source: HeartRhythm Case Rep. 2026 Mar 30;12(7):802–9. doi: 10.1016/j.hrcr.2026.03.020 (PMC13379360; doi:10.1016/j.hrcr.2026.03.020)
Supplement: Supplemental Methods [file mmc1.docx]

***KCNQ1* D673N Variant Causes Loss of Adrenergic-Induced Cardiac Action Potential Shortening**

Naheed Fatima, Lydia D. Hellwig, Michael G. Klein, Cecelia C. Mangione, Princess Nwachukwu, Joshua Salzer, Clifton L. Dalgard, Joaquin Villar, Mark C. Haigney and Thomas P. Flagg

**Expanded Methods**

**Genomic Sequencing**

This individual was enrolled in the Genetic Exploration of Military Sudden Cardiac Arrest (GEMini) Study. This study was reviewed and approved by the Uniformed Services University Institutional Review Board (MED-83-8797). Whole genome sequencing was conducted at The American Genome Center (TAGC). Sequencing was performed on an Illumina NovaSeq 6000. Primary sequencing data demultiplexing, alignment and variant calling were performed using the Illumina HAS2.2 pipeline and sample-level quality control for base quality, coverage, duplicates and contamination was conducted before variant annotation and interpretation was completed. Variants were classified according to the standards and guidelines for sequencing interpretation of the American College of Medical Genetics and Genomics (ACMG) by a clinical molecular geneticist. The individual was found to be heterozygous for a variant of uncertain significance in *KCNQ1:* c. 2017G>A. This variant was also identified and classified as a variant of uncertain significance through clinical genetic testing at GeneDx Laboratory via the Combined Cardiac Sequencing and Deletion/Duplication Panel of 139 genes.

**cDNA plasmids**

Plasmids carrying *KCNQ1*  (Addgene plasmid # 173161 ; http://n2t.net/addgene:173161; RRID:Addgene_173161) and *KCNE1* (Addgene plasmid # 173160 ; http://n2t.net/addgene:173160 ; RRID:Addgene_173160) were gifts from Al George.^1, 2^ The D673N missense variant was introduced into the KCNQ1 cDNA using overlap extension PCR. The sequence of the resultant cDNA including incorporation of the missense variant was confirmed by Sanger sequencing.

**Cell culture**

HEK293 cells were cultured in Dulbecco's Modified Eagle Medium (Gibco; cat # 11995-065) supplemented with 10% fetal bovine serum, 100 U/ml penicillin and 100 U/ml streptomycin in a humidified atmosphere at 37 °C with 5% CO_2_. Cells were transfected with cDNA plasmids using Lipofectamine3000 Transfection Reagent (Cat # L3000001, ThermoFisher Scientific) following the manufacturer’s protocol. Voltage clamp experiments to assess current were typically performed 24-48 hours after transfection.

Control ND 2.0 hiPSCs (a gift from Dr. Jizhong Zou, Director, NHLBI iPSC Core) and CRISPR edited derivatives were used in this study. hiPSCs were maintained in Essential 8 (E8; Gibco cat # A1517001) media on 6-well plates coated with growth factor-reduced Matrigel (Corning, Cat. # 356230). For passaging cells every 4 days, hiPSC were dissociated with 0.5 mM EDTA in PBS for 5-10 minutes at room temperature and seeded on Matrigel coated plated in E8 supplemented with 10 µM RHO kinase inhibitor Y-27632 (Tocris; cat # 1254). After 24 hours, the media was replaced with E8 without Y-27632. Media was replaced daily until the next passage.

**CRISPR-Cas engineering of c.2017G>A (p.D673N) variant in ND2.0 hiPSC line**

To introduce the variant in the KCNQ1 gene in the ND2.0 hiPSC line, we utilized CRISPR-Cas technology. The guide RNA (gRNA) and homology directed repair (HDR) template incorporating the VUS were designed and synthesized by Integrated DNA Technologies (IDT). The sequences of the gRNA and HDR template were:

gRNA: 5’-CCAGGAGGGGCCCCGAUGAG

HDR template: 5’-GCCCACCTACGAGCAGCTGACCGTGCCCAGGAGGGGCCCC**A**ATGAGGG**C**TCC TGAGGAGGGGATGGGGCTGGGGGATGGGCCT

The marked nucleotides in the HDR denote the VUS (*KCNQ1:* c. 2017G>A) and a silent mutation (*KCNQ1:* c. 2025G>C) that abolishes the PAM site to minimize repeated cleavage once repair has occurred.

Ribonucleoprotein (RNP) complexes were assembled by combining 0.5 µl gRNA (100 µM) with 0.3 µl Cas9 nuclease (61 µM stock; IDT, Cat #: 1081058) at room temperature. After 10 min, 1.5 µl HDR template (100 µM) and 200,000 iPSCs were added to the RNP complex in a total volume of 10 µl and electroporated (1400 V, 20 ms, 1 pulse) using the NEON Transfection system (Invitrogen). Transfected cells were grown for 2 days in matrigel coated 24 well plates followed by cell lysis, PCR and sequencing to assess incorporation of the mutant sequence. ICE analysis (Synthego) was used to determine editing efficiency. Edited cells were subjected to >3 rounds of single cell dilution to obtain a clonal population.

**Cardiomyocyte differentiation**

To obtain ventricular-type cardiomyocytes from hiPSCs, we used a method adapted from Lin, et al.^3^ Briefly, ~150,000 hiPSCs were plated per well of a 6-well plate and grown until ~90% confluency in E8 media. On Day 1, E8 was replaced with cardiomyocyte differentiation basal media (CDBM) comprised of DMEM/F12 (Gibco, Cat # 11320033) supplemented with 10mL/L fatty acids (Gibco, Cat# 11905-031), 14 ug/L sodium selenate, 64 mg/L ascorbic acid and 11 mg/L holotransferrin. Cells were exposed to a GSK3 inhibitor CHIR (5 µM) in CDBM for the first 24 hours to initiate mesenchymal induction, followed by recovery in CDBM + heparin (3 µg/mL) for 1 day. Cells were next treated with the WNT inhibitor (IWP2; 2.5 µM) and heparin (3 µg/mL) in CDBM for 72 hours followed by 3 days of recovery in CDBM + Heparin (3 µg/mL). Following 2 days of incubation in CDBM, metabolic selection was performed using RPMI (- glucose; + 10 mM D-lactate) for 4 days. Cells were maintained in CDBM after selection until used for experiments.

**Electrophysiological methods**

Ionic currents were recorded from transfected HEK293 cells in the whole cell configuration using an Axopatch 200-B amplifier coupled to a Digidata 1440A interface and controlled by PClamp 10 software (Molecular Devices, San Jose CA). Acquisition rates were 10 or 50 kHz, low-pass filtered at 5 or 10 kHz. Thick-walled micro-electrodes were fire-polished (1-3 MΩ). HEK293 cells grown on glass coverslips were transfected with either KCNQ1 WT or KCNQ1-D673N cDNA + KCNE1 + GFP, and used after 24-48 hours. The perforated-patch technique was used to avoid rapid rundown of KCNQ1-mediated current. The extracellular solution contained (mM): NaCl 140, KCl 5.4, HEPES 10, MgCl_2_ 1, CaCl_2_ 1.8, glucose 10, pH 7.4, 295-305 mOsm.^4^  The pipette solution contained: K^+^ Aspartate 125, KCl 20, NaCl 10, H+-HEPES 10, MgCl_2_ 5, K_2_-EGTA 0.1, amphotericin B 0.24, pH 7.2, 290-295 mOsm. *I*_Ks_ currents under voltage-clamp were recorded after electronic compensation of series resistance (≥ 80%) and capacitance using a depolarizing step command. The command pulse was rolled-off with an exponential time constant of 5-10 ms in order to blunt the capacitive current transient. The holding potential was -60 mV. Temperature was maintained at 35-37 ºC using an in-line solution heater and temperature controller (Warner, Holliston MA).

Action potentials paced at 1 Hz and intracellular Ca^2+^ transients were recorded in iPSC cardiomyocytes grown on coverslips at low density. Cells were loaded with the fluorescent Ca^2+^ indicator Cal-520-AM (AAT Bioquest, Pleasanton CA), 1 uM for 10 min at 37 deg C, then washed for 10 min prior to recording. Epi-illumination was via a 490 nm LED (Sutter Instruments, CA) and dichroic cube (FITC350C, Semrock) through a variable incident aperture; fluorescence was recorded simultaneously (D104 photometer, PTI, NJ) with action potentials, both arising from individual cells spatially isolated from neighbors. Illumination was limited to every *n*th beat (*n*=1-5) using an Arduino controller/counter to minimize photobleaching. Peak levels of Ca^2+^ indicator-related fluorescence achieved during recordings were found to be ≤ 50% of indicator saturation, therefore requiring no correction. Recording modality (perforated patch), extracellular and pipette solutions and recording temperature were as in HEK293 cell experiments. Drugs used to assess activity of IKs in cardiomyocytes were dissolved in extracellular solution to the indicated concentration from a 10-mM DMSO stock solution (HMR1556, ML277; Tocris/Bio-Techne, Minneapolis MN), or dissolved directly (Isoproteronol, Sigma-Aldrich).

**Multielectrode Array (MEA) Measurements**

Monolayers of hiPSC-CMs were plated on multielectrode arrays for analysis using the Maestro platform (Axion Biosystems). Briefly, cardiomyocytes were plated on the fibronectin-coated wells of a 24-well MEA plate (16 electrodes per well) at a density of 70,000 cells per well. Cells were allowed to attach for 3-4 days prior to experiments. Extracellular field potentials were measured before (Control) and 60-90 minutes after drug addition. Drugs were diluted in DMEM+4.5g/L sodium pyruvate (Sigma). The duration of the field potential (FPD) was determined as the time from the “Na^+^ spike” marking the start of depolarization and the end of the “T wave” that coincides with repolarization, using the median of a change-point detection algorithm^5^ applied to each electrode signal of a given well.

**Data and Statistical Analysis**

Electrophysiological and optical data were analysed offline using bespoke routines written in IDL software (NV5 Geospatial Systems). Ionic current amplitudes expressed as current densities (pA/pF) using the measured cell capacitance. Data are reported as means ± SEM or SD. The normality of sample distributions was determined using the Shapiro-Wilk test. Statistical differences between means were determined using a two-tailed paired or unpaired t-test for comparisons between groups.  A value of P < 0.05 was considered statistically significant.

**References**

1. Kuenze G, Vanoye CG, Desai RR, et al. Allosteric mechanism for KCNE1 modulation of KCNQ1 potassium channel activation. Elife 2020;9.

2. Vanoye CG, Desai RR, Fabre KL, et al. High-Throughput Functional Evaluation of KCNQ1 Decrypts Variants of Unknown Significance. Circ Genom Precis Med 2018;11:e002345.

3. Lin Y, Linask KL, Mallon B, et al. Heparin Promotes Cardiac Differentiation of Human Pluripotent Stem Cells in Chemically Defined Albumin-Free Medium, Enabling Consistent Manufacture of Cardiomyocytes. Stem Cells Transl Med 2017;6:527-538.

4. Dong MQ, Lau CP, Gao Z, Tseng GN, Li GR. Characterization of recombinant human cardiac KCNQ1/KCNE1 channels (I (Ks)) stably expressed in HEK 293 cells. J Membr Biol 2006;210:183-192.

5. Close RI. Activation delays in frog twitch muscle fibres. J Physiol 1981;313:81-100.
