# Supplementary material for: KCNQ1 D673N variant causes loss of adrenergic-induced cardiac action potential shortening
Source: HeartRhythm Case Rep. 2026 Mar 30;12(7):802–9. doi: 10.1016/j.hrcr.2026.03.020 (PMC13379360; doi:10.1016/j.hrcr.2026.03.020)
Supplement: Supplemental Figure 1 [file mmc2.docx]

***KCNQ1* D673N Variant Causes Loss of Adrenergic-Induced Cardiac Action Potential Shortening**

Naheed Fatima, Lydia D. Hellwig, Michael G. Klein, Cecelia C. Mangione, Princess Nwachukwu, Joshua Salzer, Clifton L. Dalgard, Joaquin Villar, Mark C. Haigney and Thomas P. Flagg

**Data Supplement**

**
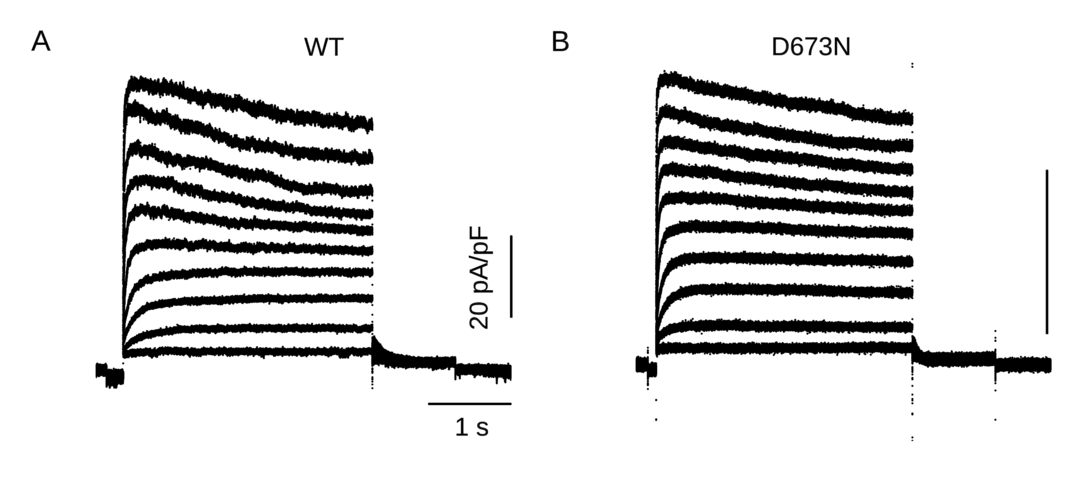
**

**Figure S1.** Family of ionic currents from HEK293 cells transfected with KCNQ1+GFP (without KCNE1) for WT (A) and D673N (B) variant. Each sweep from a test voltage between -40 and +50 mV, from a holding potential of -60 mV. Data are representative of N=4 cells of each type.
